# Digital Mindfulness Intervention for Pregnant Women With Affective Disorders and Acute Stress Reactions: Prespecified Secondary Analysis of a Randomized Controlled Trial

**DOI:** 10.2196/93811

**Published:** 2026-09-02

**Authors:** Kathrin Haßdenteufel, Mitho Müller, Jan Multmeier, Harald Abele, Sara Yvonne Brucker, Stefan Zipfel, Johanna Graf, Armin Bauer, Peter Jakubowski, Jan Pauluschke-Fröhlich, Markus Wallwiener, Stephanie Wallwiener

**Affiliations:** 1 Department of Gynecologic Endocrinology and Fertility Disorders, University Hospital Heidelberg, Im Neuenheimer Feld 440, Heidelberg, Baden-Wurttemberg, 69120, Germany, 49 062215637021; 2 Department of Psychology, Ludwig-Maximilians- University, Munich, Germany; 3 Freie Universität Berlin, Berlin, State of Berlin, Germany; 4 Department of Women’s Health, University Hospital Tübingen, Tübingen, Germany; 5 Department of Psychosomatic Medicine and Psychotherapy, University Hospital Tübingen, Tübingen, Germany; 6 Clinic for Psychosomatic Medicine and Psychotherapy, LVR-University Hospital Essen, University of Duisburg-Essen, Essen, Germany; 7 Clinic für Psychosomatic Medicine and Psychotherapy, Klinikum Stuttgart, Stuttgart, Germany; 8 Department of Women’s Health, Research Institute for Women's Health, University Hospital Tübingen, Tübingen, Germany; 9 Department of Gynecology, University Hospital Halle, Halle, Saxony-Anhalt, Germany; 10 Department of Obstetrics and Perinatal Medicine, University Hospital Halle, Halle, Saxony-Anhalt, Germany

**Keywords:** mindfulness, pregnancy, mental disorder, digital intervention, depression, anxiety

## Abstract

**Background:**

Pregnant women with *ICD-10* (*International Statistical Classification of Diseases, Tenth Revision*) affective or stress-related disorders face an elevated risk of perinatal depression and anxiety, yet evidence on digital nonpharmacologic interventions for this population remains limited.

**Objective:**

This study evaluated the effectiveness of an 8-week digital mindfulness–based intervention (eMBI) compared with treatment as usual (TAU) among pregnant women with *ICD-10* affective or stress-related disorders participating in a randomized controlled trial (RCT).

**Methods:**

This prespecified secondary analysis was conducted within a multicenter RCT in Baden-Württemberg, Germany. Pregnant women aged 18 years and older with elevated depressive symptoms (Edinburgh Postnatal Depression Scale [EPDS]>9) and *ICD-10*–diagnosed affective or stress-related disorders were randomized 1:1 to eMBI or TAU. The intervention consisted of 8 weekly app-based mindfulness sessions (45 min each) delivered during gestational weeks 29‐36, with no direct therapist contact. The primary outcome was continuous depressive symptom severity measured with the EPDS at 4‐6 weeks post partum. Secondary outcomes included the EPDS at 6 months post partum, generalized anxiety (State-Trait Anxiety Inventory-State [STAI-S], State-Trait Anxiety Inventory-Trait [STAI-T]), and Pregnancy-Related Anxiety Questionnaire-Revised (PRAQ-R). Analyses followed the intention-to-treat (ITT) principle, using mixed models for repeated measures and multiple imputation.

**Results:**

Of the 5299 screened women, 147 met the inclusion criteria for this subgroup analysis (intervention group [IG] had n=73 women and control group had n=74 women). Groups were comparable at baseline. The IG showed significantly greater reductions in EPDS scores at gestational week 34 (Δ=–2.21, *P*=.01), week 36 (Δ=–3.25, *P*=.01), and 4‐6 weeks post partum (Δ=–4.81, *P*=.007). Treatment effects remained robust under conservative missing-data assumptions. At 4-6 weeks post partum, a higher proportion of participants in the IG achieved clinically meaningful improvement (31/73, 42.5% vs 21/74, 28.4%; adjusted odds ratio 1.56, 95% CI 1.19‐2.05; *P*=.001). Anxiety outcomes followed a similar pattern, whereas pregnancy-related anxiety did not differ between groups.

**Conclusions:**

In this prespecified subgroup of pregnant women with *ICD-10* affective or stress-related disorders, the eMBI was associated with clinically meaningful reductions in depressive symptoms from late pregnancy to 4-6 weeks post partum. Effects at 6 months post partum were attenuated and less stable across missing-data assumptions. These findings support eMBIs as a scalable, nonpharmacological adjunct to perinatal mental health care for women with affective or stress-related disorders, while confirmation in adequately powered trials with strategies to reduce postpartum attrition is warranted.

## Introduction

Pregnancy and early post partum are periods during which women are particularly susceptible to mental health disorders such as anxiety, depression, and acute stress reactions, affecting up to one-quarter of all pregnant women [1,2]. Women who have had previous experiences with anxiety or depression, either during a previous pregnancy or independent of a pregnancy, may face an elevated risk of relapse in the perinatal phase [3]. Early postpartum mental health outcomes, particularly within the first 4‐6 weeks after birth, are clinically critical because this period marks the highest risk window for the onset or recurrence of postpartum depression. Symptom expression during this phase is strongly shaped by hormonal shifts, acute stress adaptation, and early caregiving demands, making it a sensitive indicator of perinatal vulnerability and treatment responsiveness.

Maternal depression has been linked to impaired quality of life and psychological distress, resulting in suicidal tendencies [4-6]. Moreover, medical complications surrounding the birth and postnatal phases have been reported frequently [7,8]. The infant of a mother with a psychological disorder may exhibit low birth weight, be born prematurely, and experience both neuronal and cognitive developmental impairments after birth [9,10].

Mindfulness-based approaches have been successfully used to alleviate the symptoms of perinatal anxiety and depression, along with their detrimental effects, as well as to diminish the likelihood of their recurrence [11-13]. Such approaches may involve telephone-based coaching [14], online interventions [15,16], and cognitive therapy with personal coaching [17,18]. These programs have proven to be effective in reducing depression and anxiety during the perinatal phase [19].

Although pharmacological treatment options exist, their applicability in the perinatal period is restricted. Recently, Zuranolone has been approved as the first oral agent specifically indicated for postpartum depression in both the United States and Europe. However, its use is not recommended during breastfeeding, limiting its use during a period when many women are most vulnerable to depressive symptoms [20]. This emphasizes the importance of scalable and safe nonpharmacological interventions such as mindfulness-based digital programs. While different mindfulness interventions for pregnant women have been conducted and proven effective, it is currently unknown which women could particularly benefit from such an intervention and whether the effectiveness may be hindered by psychological comorbidities among certain women [21,22]. However, there is still paucity regarding a potential impact of coexisting mental disorders on the efficacy of a digital mindfulness intervention during pregnancy and post partum in terms of depression, general anxiety, and birth-related anxiety. Therefore, this prespecified secondary analysis evaluated the effectiveness of the intervention specifically among pregnant women with elevated depressive symptoms (Edinburgh Postnatal Depression Scale [EPDS]>9) who also had an *ICD-10 (International Statistical Classification of Diseases, Tenth Revision)*–diagnosed affective or stress-related disorder [23].

## Methods

### Study Design, Participants, and Intervention

This prospective study is a prespecified secondary analysis of the multicenter randomized controlled trial (RCT) conducted as a part of the Innovation Fund Project Mind:Pregnancy (01NVF17034) in the state of Baden-Württemberg, Germany. Participating centers included the university hospitals of Heidelberg and Tübingen, as well as more than 200 gynecological practices, as previously described and published [19]. Initial screening for eligibility took place routinely from February 2019 to October 2020 with 5299 pregnant women and was conducted using the EPDS. Women with subclinical signs of depression scoring more than 9 were invited to participate in the study. The patients underwent a diagnostic assessment and psychological evaluation by a trained psychologist. A short version of the Structured Clinical Interview for the *Diagnostic and Statistical Manual of Mental Disorders, Fifth Edition-*Clinician Version (SCID-5-CV), was used to exclude acute needs for psychological treatment, including acute psychotic episodes, acute bipolar affective symptoms, acute substance abuse, borderline personality disorder, and acute traumatic events without a relation to the current pregnancy.

Additional inclusion criteria encompassed being 18 years of age or older, adequate proficiency in German, a singleton pregnancy, no known neonatal malformations or anomalies, less than 29 weeks of gestation at screening, having health insurance coverage through one of the participating statutory health insurance providers, and residing within the state of Baden-Württemberg. The exclusion criteria have been described in detail in the previously published study protocol [19].

### Intervention

Study participants were randomly assigned at a ratio of 1:1 to the intervention group (IG) or the control group (CG). The IG was given access to the digital mindfulness–based intervention (eMBI) with 8 weekly sessions during weeks 29 to 36 of pregnancy, lasting 45 minutes, whereas the CG received treatment as usual (TAU) without any restrictions or regulations within the study period. TAU referred to routine obstetric and mental health care available outside the study. Participants in the CG did not receive any additional study-specific psychological intervention, mindfulness training, or therapist contact through the study. The use of external pharmacological or nonpharmacological treatment was not restricted and was documented where available.

Both groups completed the questionnaires digitally via an application and had access to an online pregnancy guidebook. For further information about the intervention, see the study protocol [19].

### Prespecified Secondary Subgroup Analysis

This prespecified secondary analysis was restricted to participants from the parent RCT who had elevated depressive symptoms at screening (EPDS>9) and an *ICD-10*–diagnosed affective or stress-related disorder at diagnostic assessment. The analyzed sample included 147 participants, of whom 73 were allocated to the IG and 74 to the TAU-CG. Participants without an *ICD-10* affective or stress-related disorder were part of the parent trial but were not included in the present analysis.

Diagnoses included depressive disorders (*ICD-10*: F32-F33), anxiety disorders (F40-F41), somatoform or dissociative disorders (F44-F45, F48.1, and F68.0), and stress-related disorders or acute stress reactions (F43 and F62). The present analysis compared outcomes between the IG and CG within this diagnostically defined subgroup.

### Assessment Schedule

Assessments were conducted digitally at predefined time points from pregnancy to postpartum follow-up. Baseline information, diagnostic status, and sociodemographic characteristics were assessed before randomization. Depressive symptoms were assessed using the EPDS, generalized anxiety using the State-Trait Anxiety Inventory-State (STAI-S) and State-Trait Anxiety Inventory-Trait (STAI-T), and pregnancy-related anxiety using the Pregnancy-Related Anxiety Questionnaire-Revised (PRAQ-R). The main antenatal follow-up assessments were conducted during late pregnancy, including gestational week 34 (T4) and gestational week 36 (T5). Postpartum follow-up assessments were conducted at 4‐6 weeks post partum (T6) and 6 months post partum (T7). The primary outcome was EPDS symptom severity at T6.

The extent of missing data was summarized descriptively by group, time point, and outcome. Missingness patterns were examined to identify whether missing values increased over time and whether they differed between the study groups.

### Outcome Variables

#### Primary Outcome

The EPDS, which was originally developed by Cox et al [24] and translated into German by Bergant et al [25], is a 10-item self-rating scale that assesses depressive symptoms during the peripartum. The questionnaire encompasses 10 items measuring depressive symptom severity on a 4-point Likert scale (0‐3) within the past 7 days, producing a total score ranging between 0 and 30. Higher scores indicate higher levels of depression. The EPDS has proven to be a valid and sensitive instrument for predicting depressive disorders not only in the postnatal period but also antenatally [26]. The most commonly used cutoff value of 9 (EPDS>9) has shown a sensitivity of 0.96 and specificity of 1.00 in former research [27]. The primary outcome was depressive symptom severity measured continuously with the EPDS at 4-6 weeks post partum (T6). The EPDS>9 threshold was used as a screening criterion for eligibility in the parent trial and should not be interpreted as the definition of the primary outcome. In the primary analysis, EPDS total scores were analyzed as continuous outcomes.

#### Secondary Outcomes

##### State-Trait Anxiety Inventory

Anxiety was measured using the German version of the State-Trait Anxiety Inventory (STAI) during the perinatal period. The questionnaire consists of 2 scales: the STAI-S (state scale), which evaluates anxiety as a temporary condition, encompassing feelings of tension, nervousness, and worry, and the STAI-T (trait scale), which refers to dispositional anxiety over time. Each of the 20 items is measured using a 1 (not at all) to 4 (very much) Likert scale. The sum, ranging from 20 to 80, indicates the magnitude of situational and dispositional anxiety [28].

##### PRAQ-R

The PRAQ-R is an abridged 10-item version of a self-report instrument assessing pregnancy- and birth-specific anxiety and is a valid predictor of birth and childhood outcomes [29-31]. The questionnaire encompasses fear of giving birth, worries about bearing a physically or mentally handicapped child, and concerns about one’s own appearance [32]. Item responses were summed to derive a total PRAQ-R score, with higher scores indicating higher levels of pregnancy-related anxiety.

### Statistical Analysis

#### Multiple Imputation and Sensitivity Analyses

All analyses followed the intention-to-treat (ITT) principle. Missing data were handled using multiple imputation by chained equations (MICE), generating 5 imputed datasets. Missing values were estimated based on the observed data under the assumption of missing at random (MAR). Imputation was performed for the following end points: EPDS, STAI-T and STAI-S, and PRAQ-R. This approach increases statistical power and reduces the risk of bias due to dropout. The baseline EPDS score was included as a covariate in all adjusted logistic regression models to account for heterogeneity in depressive symptom severity among participants meeting the EPDS>9 screening threshold.

For sensitivity analyses, 2 reference-based multiple imputation approaches were applied. Under the copy-increments-in-reference (CIR) assumption, missing postdropout values in the IG were imputed by applying the observed change trajectory of the CG while preserving the treatment effect achieved up to dropout. Under the jump-to-reference (J2R) assumption, participants in the IG with missing follow-up data were assumed to follow the outcome trajectory of the CG after dropout, thereby setting any postdropout intervention effect to 0.

The extent of missing data was summarized descriptively by treatment group, time point, and outcome. We examined whether missing values increased over time and whether the proportion of missingness differed between the IG and CG. Missingness was reported separately for the continuous outcomes and for the derived binary response and remission outcomes. Detailed proportions of missing data are provided in Multimedia Appendix 1.

The primary outcome was analyzed using mixed models for repeated measures (MMRM), including the baseline value of the respective end point, group, time, and the group × time interaction as fixed effects, with subject-specific random intercepts. Estimated marginal means for each time point and between-group differences were reported with 95% CIs. In addition, effect sizes were calculated as Cohen *d* with corresponding 95% CIs.

#### Responder and Remission Analyses

To assess clinical relevance, response and remission were analyzed as binary outcomes. Response was defined as a reduction of at least 4 points in the EPDS score from baseline to the respective follow-up assessment. Remission was defined as an EPDS score of 9 and higher at the respective follow-up assessment. Descriptive rates were calculated as the number of participants meeting the respective criterion divided by the total number of participants randomized to the corresponding group, with missing values reported as a separate category. For response and remission outcomes, logistic regression models were fitted using observed cases at the respective follow-up assessment. The treatment group was included as the main independent variable, and the baseline EPDS score was included as the only covariate. No additional covariates were included in these models. Odds ratios (ORs) with 95% CIs were reported. Because these analyses were based on observed cases, they were considered supportive analyses of clinical relevance. The logistic regression models were based on participants with available data for all variables required for the respective model, namely treatment group, baseline EPDS score, and the derived binary response or remission outcome at the corresponding follow-up assessment.

To explore the robustness of the findings, per-protocol analyses were additionally conducted, including only participants who adhered to the study protocol without major deviations.

All statistical analyses were conducted using SPSS (version 29.0; IBM Corp) and R (version 4.2; R Foundation for Statistical Computing). A 2-sided significance level of *P*<.05 was applied.

### Ethical Considerations

The study protocol was approved by the ethics committee of the Medical Faculty of the Universities of Heidelberg (S-744/2018) and Tübingen (952/2018BO2), Germany, and was conducted in accordance with the Declaration of Helsinki and its subsequent amendments. All personal data were collected and processed subject to confidentiality and the European General Data Protection Regulation (EU-GDPR) [33]. All participants provided signed written consent and received €100 (1€=US $1.14 as of July 27, 2026) each as financial compensation. The study participants had the right to request information about the personal data collected from them. Participation in the study was voluntary after all study content and objectives had been declared and written consent had been given; participation could be withdrawn at any time without any disadvantages for the patient. Patient data and clinical data were recorded pseudonymously and stored centrally in an electronic case report form (eCRF). This study follows the CONSORT (Consolidated Standards of Reporting Trials; Checklist 1 [34]) statement and the SPIRIT (Standard Protocol Items: Recommendations for Intervention Trials [35]) guidelines. The study was registered with the German Clinical Trials Registry (DRKS00017210).

## Results

### Descriptive Characteristics

**Figure 1.**
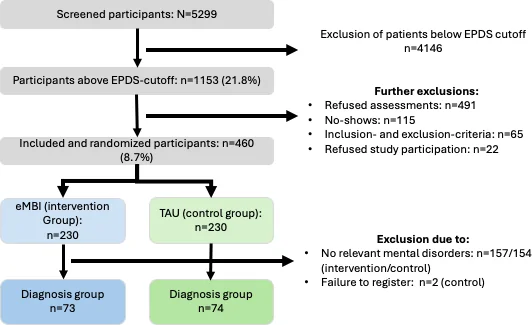
Flow diagram of participant screening, eligibility assessment, randomization, and inclusion in the secondary analysis. eMBI: digital mindfulness–based intervention; EPDS: Edinburgh Postnatal Depression Scale; TAU: treatment as usual.

A total of 147 participants were included in the analysis, with 74 women in the CG and 73 in the IG (Figure 1). Baseline demographic and socioeconomic characteristics were broadly comparable between the groups. For example, maternal age, parity, educational level, partnership status, and baseline EPDS scores showed no clinically relevant between-group imbalances (Table S1 in Multimedia Appendix 1).

The most frequently documented primary diagnoses were recurrent depressive disorders (*ICD-10*: F32-F33), anxiety disorders (F40-F41), and stress-related disorders (F43). The distribution of these diagnostic groups was comparable between the IG and CG (Table 1).

**Table 1.** Distribution of *ICD-10^a^* diagnostic categories in the analyzed subgroup by treatment group.

Diagnosis (*ICD-10*)	Control group (n=74), n (%)	Intervention group (n=73), n (%)
Depressive disorders (F32-F33)	33 (44.6)	29 (39.7)
Anxiety disorders (F40-F41)	19 (25.7)	19 (26.0)
Somatoform and dissociative disorders (F44-F45; F48.1; F68)	1 (1.4)	1 (1.4)
Acute stress reactions (F43)	20 (27.0)	22 (30.1)
Outcome of delivery (Z73)	1 (1.4)	2 (2.7)

a *ICD-10*: *International Statistical Classification of Diseases, Tenth Revision*.

### Primary Outcome

#### Depressive Symptoms (EPDS)

In relation to the EPDS, a higher proportion of missing values can be observed in the IG (Table S2 in Multimedia Appendix 1). Moreover, the proportion of missing values increases substantially toward the end of the study period (T6 and T7), such that at T6, 40.5% (30/74, CG) and 47.9% (35/73, IG) of EPDS values were not observed.

**Figure 2.**
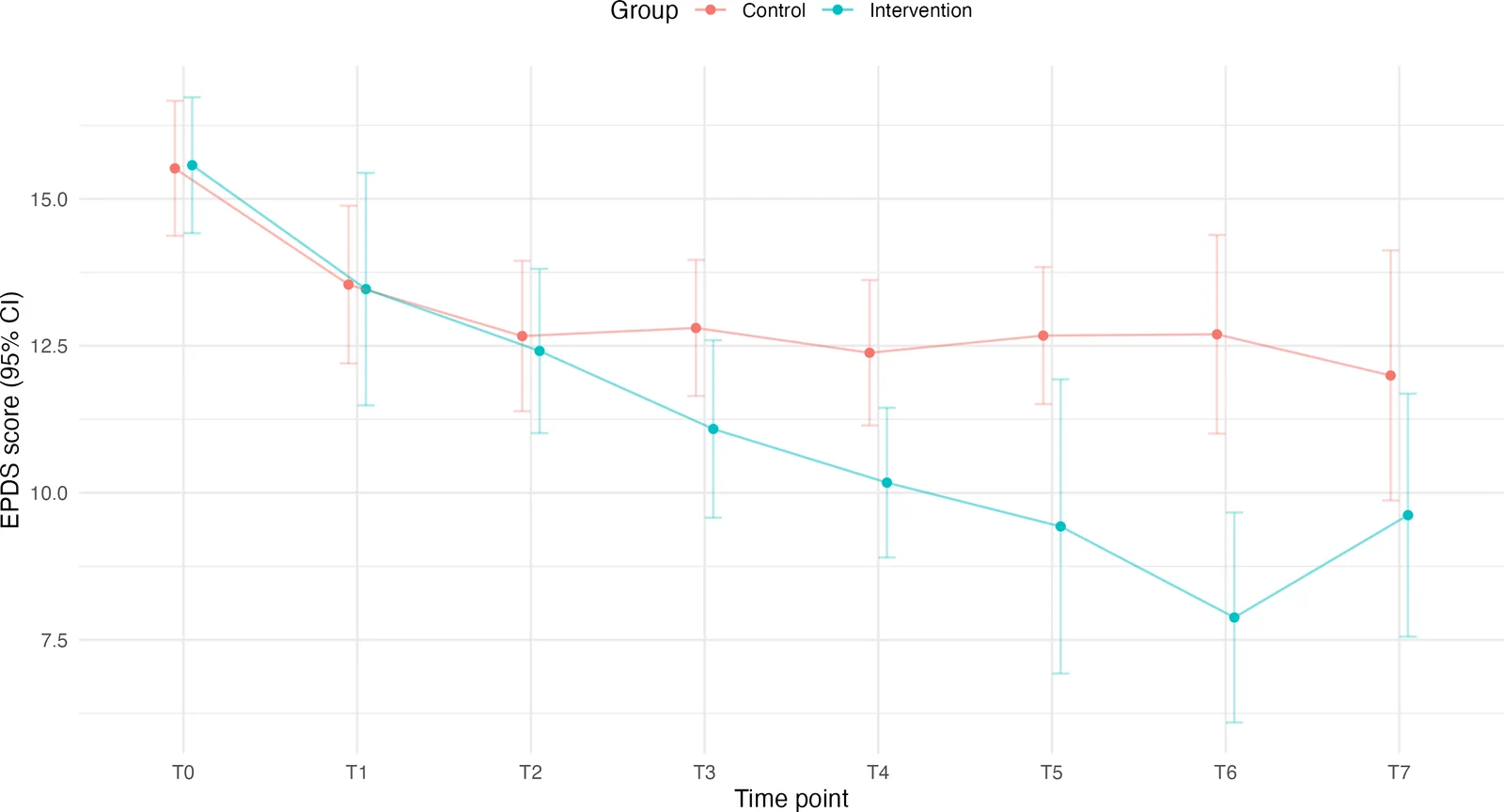
Multiple imputation by chained equations (MICE)–imputation for Edinburgh Postnatal Depression Scale (EPDS) scores over time.

In the primary analysis based on MICE-imputed data, significant advantages for the IG were observed at time points T4 (difference=2.21, *P*=.01, *d*=0.57, 95% CI 0.12‐1.03), T5 (difference=3.25, *P*=.01, *d*=0.84, 95% CI 0.13‐1.55), and T6 (difference=4.81, *P*=.007, *d*=1.25, 95% CI 0.47‐2.03). All other effects were not statistically significant (*P*>.05; Figure 2 and Table S3 in Multimedia Appendix 1).

#### Estimated Marginal Means: MICE-Imputed Data

Assuming systematically missing EPDS values and applying CIR—that is, imputing missing data in the IG based on the observed trajectory of the CG—the intervention effects remained significant at the following time points: T4 with a difference=1.97, *P*=.05, and *d*=0.51 (95% CI 0.00‐1.02); T5 with a difference=2.47, *P*=.02, and *d*=0.64 (95% CI 0.09‐1.19); and T6 with a difference=3.01, *P*=.003, and *d*=0.78 (95% CI 0.23‐1.33).

Under the J2R assumption, whereby participants in the IG who discontinued follow-up were assumed to return to the outcome trajectory of the CG, the intervention effects remained significant at the following time points: T4 with a difference=1.99, *P*=.03, and *d*=0.52 (95% CI 0.06‐0.98); T5 with a difference=2.65, *P*=.005, and *d*=0.69 (95% CI 0.21‐1.17); and T6 with a difference=2.51, *P*=.03, and *d*=0.65 (95% CI 0.07‐1.23).

These findings confirm the robustness of the intervention effects even under conservative assumptions of nonrandom missingness. Thus, the results were robust under conservative missing-data assumptions.

Missing outcome data increased over the course of follow-up, with the highest proportions observed during the postpartum assessments. For the EPDS at T6, values were missing for 40.5% (30/74) of participants in the CG and 47.9% (35/73) of participants in the IG. At T5, missing EPDS values were observed in 8.1% (6/74) of the CG and 35.6% (26/73) of the IG. This pattern indicates increasing missingness over time and a higher proportion of missing EPDS data in the IG at later assessments. Detailed proportions of missingness by outcome, time point, and treatment group are shown in Table S2 in Multimedia Appendix 1.

#### Response Rates

Descriptive analyses showed that the proportion of responders increased steadily in the IG throughout pregnancy, reaching the highest values around T4-T5, and remained higher than in the CG post partum. At T5, the proportion of participants with a response was 47.3% (35/74) in the CG and 46.6% (34/73) in the IG. However, a substantial proportion of missing values was observed in the IG (26/73, 35.6%), which limits the interpretability of the data. At T6, descriptively, a higher proportion of responders was found in the IG (31/73, 42.5% vs 21/74, 28.4%). Response and remission outcomes were analyzed as predefined binary outcomes derived from the EPDS, as described in the *Methods* section. At T6, response was observed in 42.5% (31/73) of participants in the IG and 28.4% (21/74) of participants in the CG. Remission was observed in 36.9% (27/73) and 21.6% (16/74), respectively. At this time point as well, a large proportion of values was missing in both groups. The results are summarized in Table 2.

**Table 2.** Edinburgh Postnatal Depression Scale (EPDS) response status at 36 weeks of gestation and 4‐6 weeks post partum by treatment group.^a^

EPDS response status^b^	Control group, n=74	Intervention group, n=73
T5^c^, n (%)		
Improved	35 (47.3)	34 (46.6)
Unchanged	23 (31.1)	10 (13.7)
Worsened	10 (13.5)	3 (4.1)
Missing	6 (8.1)	26 (35.6)
T6^d^, n (%)		
Improved	21 (28.4)	31 (42.5)
Unchanged	14 (18.9)	6 (8.2)
Worsened	9 (12.2)	1 (1.4)
Missing	30 (40.5)	35 (47.9)

a Percentages are based on all randomized participants in the respective group; missing values are reported as a separate category.

b Response was defined as a reduction of 4 and higher EPDS points from baseline.

c T5: 36 weeks of gestation.

d T6: 4-6 weeks post partum.

In a baseline-adjusted logistic regression model based on observed cases, the IG showed higher odds of response at T6 than the CG (OR 1.56, 95% CI 1.19‐2.05; *P*=.001). Because descriptive rates were calculated using all randomized participants as the denominator, with missing values reported separately, whereas the logistic regression model included only participants with available outcome data, these estimates should be interpreted as supportive indicators of clinical relevance rather than definitive efficacy estimates.

#### Remission Rates

A similar pattern was observed for remission. At T5 (36 wk of gestation), 35.6% (26/73) of women in the IG reached remission, compared to 32.4% (24/74) in the CG. At T6, remission rates diverged more strongly, with 36.9% (27/73) in the IG compared with 21.6% (16/74) in the CG (Table 3).

**Table 3.** Edinburgh Postnatal Depression Scale (EPDS) remission status at 36 weeks of gestation and 4‐6 weeks post partum by treatment group.^a^

EPDS remission status^b^	Control group, n=74	Intervention group, n=73
T5^c^, n (%)		
Remission	24 (32.4)	26 (35.6)
No remission	44 (59.5)	21 (28.8)
Missing	6 (8.1)	26 (35.6)
T6^d^, n (%)		
Remission	16 (21.6)	27 (36.9)
No remission	28 (37.8)	11 (15.1)
Missing	30 (40.5)	35 (47.9)

a Percentages are based on all randomized participants in the respective group; missing values are reported as a separate category.

b Remission was defined as EPDS≤9.

c T5: 36 weeks of gestation.

d T6: 4-6 weeks post partum.

#### Logistic Regression

Baseline-adjusted logistic regression analyses confirmed that, at T5, the odds of both response (OR 1.57, 95% CI 1.20-2.06; *P*<.001) and remission (OR 1.99, 95% CI 1.49‐2.66; *P*<.001) were significantly higher in the IG than in the CG.

These findings demonstrate that the intervention not only reduced mean EPDS scores but also led to clinically meaningful improvements in a substantially higher proportion of participants. However, because this analysis was restricted to participants with available baseline and follow-up EPDS data, the estimates should be interpreted cautiously in light of the substantial and differential missingness at postpartum follow-up.

### Secondary Outcomes

Secondary end points included generalized anxiety symptoms assessed using the STAI-T (trait component) and STAI-S (state component), as well as pregnancy-specific anxiety symptoms assessed using the Pregnancy-Related Anxiety Questionnaire (PRAQ).

#### STAI-T

For STAI-T, significant effects were observed at T4 (difference=5.25, *P*=.004, *d*=0.57, 95% CI 0.17‐0.97) and T6 (difference=6.07, *P*=.03, *d*=0.66, 95% CI 0.09‐1.23; Table S4 in Multimedia Appendix 1).

In a sensitivity analysis with CIR-imputed data, the positive effect at T6 remained as a tendency but was no longer statistically significant (difference=3.48, *P*=.08, *d*=0.37, 95% CI –0.05 to 0.79). At T4, a statistically significant effect was still present (difference=3.95, *P*=.02, *d*=0.42, 95% CI 0.06‐0.78).

Under J2R imputation, the between-group effect at T6 was further reduced and not statistically significant (difference=1.99, *P*=.34, *d*=0.21, 95% CI –0.23 to 0.66), whereas the effect at T4 persisted (difference=3.78, *P*=.03, *d*=0.40, 95% CI 0.04‐0.77).

#### STAI-S

For the STAI-S, significantly lower anxiety symptoms were observed in the IG at T4 (difference=4.39, *P*=.04, *d*=0.42, 95% CI 0.02‐0.82), T5 (difference=4.71, *P*=.01, *d*=0.45, 95% CI 0.09‐0.81), and T6 (difference=5.63, *P*=.003, *d*=0.54, 95% CI 0.15‐0.92; Table S5 in Multimedia Appendix 1).

Under the assumption of CIR-imputed data, the effects at T4 (difference=2.82, *P*=.17, d=0.27, 95% CI –0.12 to 0.66), T5 (difference=3.97, *P*=.06, *d*=0.38, 95% CI –0.01 to 0.78), and T6 (difference=4.12, *P*=.08, *d*=0.40, 95% CI –0.05 to 0.84) also remained as a tendency but were no longer statistically significant.

In the sensitivity analysis with J2R-imputed data, the effect at T4 remained statistically significant (difference=3.78, *P*=.03, *d*=0.40, 95% CI 0.04‐0.77), whereas the effects at T5 (difference=2.60, *P*=.15, *d*=0.28, 95% CI –0.10 to 0.65) and T6 (difference=1.99, *P*=.34, *d*=0.21, 95% CI –0.23 to 0.66) were no longer significant.

#### Pregnancy-Related Anxiety

For pregnancy-related anxiety, no significant group differences were detected at any time point in either the ITT or sensitivity analyses. Scores decreased over time in both groups, reflecting a general decline in pregnancy-specific fears toward the end of gestation (Table S6 in Multimedia Appendix 1). In the sensitivity analyses, the same pattern was observed (all *P*>.05).

Missing outcome data increased over the course of follow-up, with the highest proportions observed during postpartum assessments. For the EPDS at T6, values were missing for 40.5% (30/74) of participants in the CG and 47.9% (35/73) in the IG. Detailed proportions of missing data by outcome, time point, and group are provided in Table S2 in Multimedia Appendix 1.

## Discussion

### Principal Findings

In this prespecified secondary analysis of pregnant women with elevated depressive symptoms and *ICD-10*–diagnosed affective or stress-related disorders, allocation to the eMBI was associated with greater reductions in depressive symptoms than TAU. The most consistent effects were observed during late pregnancy and at 4-6 weeks post partum, with the latter representing the time point with the highest incidence of postpartum depression [36]. Response and remission outcomes supported the clinical relevance of these findings, although substantial postpartum missingness and attenuation of effects at 6 months post partum warrant cautious interpretation.

Regarding anxiety symptoms assessed with the STAI-T and STAI-S, the IG demonstrated significantly lower symptom severity at T4 and T6 compared to the CG. This highlights the clinical significance of the intervention, as the early postpartum phase constitutes a critical window for both the prevention and treatment of maternal mental health disorders. The anxiety findings also suggest that the intervention may have had a more immediate effect on situational anxiety than on more stable dispositional anxiety. STAI-S reflects temporary feelings of tension, nervousness, and worry, whereas STAI-T captures a more stable tendency toward anxiety. The more consistent pattern for state anxiety is plausible, as mindfulness-based exercises may primarily support momentary emotion regulation and stress reduction. Changes in trait anxiety may require longer intervention exposure, repeated practice, or additional therapeutic components.

Although the between-group differences at T7 were no longer statistically significant, this time point reflects a considerably longer interval after the intervention, during which treatment effects may naturally attenuate as postpartum environmental and psychosocial factors increasingly dominate symptom trajectories. Thus, the attenuation of effects at 6 months post partum suggests that an 8-week antenatal intervention may not be sufficient as a stand-alone approach for women with preexisting affective or stress-related disorders. The transition into the postpartum period is accompanied by changing sleep patterns, caregiving demands, and evolving psychosocial stressors, which may reduce the durability of effects achieved during pregnancy. Future digital interventions may, therefore, benefit from a maintenance phase, postpartum booster sessions, or optional stepped-care components that provide additional support after childbirth.

The importance of our study lies in the demonstrated benefits of a digital intervention especially for predisposed women at risk for adverse mental health outcomes, which has not been assessed before in the context of digital mindfulness interventions during pregnancy. It is feasible to assume that a coexisting mental disorder diagnosis contributed to the effectiveness of the digital intervention in the long-term improvement of maternal mental health. The importance of a mindfulness-based intervention for the reduction of depressive and anxiety-related symptoms is highlighted by our results, as significant improvements were observed beyond the use of the digital application [13,37].

The present findings are consistent with prior work demonstrating similar efficacy for such electronic versions of mindfulness training [38-40]. It is known that a digital approach for health interventions may be effective for patients as depression treatment [41], older patients with anxiety or depression symptoms [42], pregnant women experiencing intimate partner victimization [22], and patients with comorbid depression and substance abuse [43]. The necessity of an adaptation to the individual needs of the patient and of personal interaction with a health care professional or psychologist has been pointed out [42]. Our results are in line with the findings of Zemestani and Fazeli Nikoo [21] in that a mindfulness intervention improves both mindfulness and symptoms of mental disorders. However, our study extends existing research by including a significantly larger sample size and incorporating a digital intervention.

Overall, our findings provide clear evidence of the benefits of the intervention compared to routine care and are consistent with existing international treatment recommendations. The Canadian Network for Mood and Anxiety Treatments (CANMAT) guidelines for the management of perinatal depression explicitly recommend the use of psychosocial and psychological interventions, including mindfulness-based approaches, as first-line treatment options for mild to moderate cases, emphasizing their safety and acceptability in pregnancy and postpartum populations [44]. Zuranolone has recently become available as an oral pharmacological option specifically indicated for postpartum depression. This development expands postpartum treatment options but does not address the same clinical context as an antenatal eMBI. Because the present study did not compare eMBI with pharmacological treatment, our findings should not be interpreted as evidence of superiority over medication. Instead, they suggest that digital mindfulness–based support may serve as a scalable nonpharmacological adjunct within stepped perinatal mental health care [20].

Strengths of the study have already been published and include the prospective, longitudinal design with a follow-up period of up to 5 months after childbirth, the multidimensional approach, the broad range of confounders taken into account, and the large number of screened and included patients contributing to the high power of the RCT [42]. Associated diagnoses were made based on *DSM (Diagnostic and Statistical Manual of Mental Disorders*) and *ICD* criteria, and a broad range of confounders was taken into account. The large number of screened and included patients contributes to the high power of our RCT while comparable other research in many cases lacks an active CG [45]. Importantly, both pharmacological and nonpharmacological treatments were systematically assessed, thereby minimizing the risk of bias due to unmeasured concomitant therapies.

### Limitations

Nevertheless, some limitations must be considered. Although pharmacological treatments were recorded, residual confounding by socioeconomic or environmental variables cannot be excluded. Moreover, due to the relatively low prevalence of certain mental health diagnoses, the impact of less common disorders on treatment efficacy could not be systematically evaluated. Furthermore, adjustment for baseline EPDS scores cannot fully account for clinical heterogeneity, including differences in disorder chronicity, recurrence, comorbidity, or prior treatment history. Future research should address these factors, include larger diagnostic subgroups, and explore the long-term sustainability of intervention effects beyond the early postpartum period. At T7, effects were no longer statistically significant, which must be interpreted in light of the substantial temporal distance from the intervention period, during which declining adherence, competing postpartum demands, and nonspecific recovery processes may have diluted detectable group differences.

A further limitation is the substantial proportion of missing outcome data during the postpartum assessments, particularly in the IG. At T6, EPDS values were missing for 47.9% (35/73) of participants in the IG and 40.5% (30/74) in the CG. Although sensitivity analyses using CIR and J2R assumptions supported the robustness of the short-term effects, the higher dropout rate in the IG requires cautious interpretation. Because no formal reasons-for-dropout analysis was available, we cannot determine whether the missingness reflected general postpartum burden, reduced motivation to complete app-based assessments after the intervention had ended, perceived lack of need following symptom improvement, or friction caused by the absence of direct therapist contact. Furthermore, the observed-case responder and remission analyses may be susceptible to selection bias because participants with missing postpartum EPDS data were excluded from the logistic models; consequently, the estimated ORs may overestimate clinical benefit if missingness was related to symptom severity, treatment response, or postpartum burden.

Future studies should systematically collect reasons for dropout and app engagement metrics to clarify whether digital interventions become burdensome during the early caregiving phase.

### Conclusions

In pregnant women with *ICD-10* affective or stress-related disorders, an eMBI was associated with meaningful reductions in depressive symptoms, with the most reliable improvements observed during late pregnancy and at 4-6 weeks post partum—the clinical period of highest vulnerability to perinatal depression. Anxiety symptoms also improved but with less consistency, and effects at 6 months post partum were attenuated.

Taken together, our study provides robust evidence that digital mindfulness–based programs may serve as an effective, guideline-concordant adjunct to existing therapeutic strategies, with particular benefits for women at elevated risk due to preexisting psychiatric diagnoses. The improvements were most pronounced during late pregnancy and the early postpartum period, when women are particularly vulnerable to developing postpartum depression. These findings provide strong support for integrating scalable, digital mindfulness–based programs into perinatal mental health care in line with Canadian and European guideline recommendations.

## Supplementary material

10.2196/93811 Multimedia Appendix 1 Supplementary tables presenting baseline characteristics, missing data, imputation analyses, and pregnancy-related anxiety outcomes.

10.2196/93811 Checklist 1 CONSORT-EHEALTH checklist (V1.6.1).

## Abbreviations

CANMAT: Canadian Network for Mood and Anxiety Treatments
CG: control group
CIR: copy-increments-in-reference
CONSORT: Consolidated Standards of Reporting Trials
*DSM*: *Diagnostic and Statistical Manual of Mental Disorders*
eCRF: electronic case report form
eMBI: digital mindfulness–based intervention
EPDS: Edinburgh Postnatal Depression Scale
EU-GDPR: European General Data Protection Regulation
*ICD-10*: *International Statistical Classification of Diseases, Tenth Revision*
IG: intervention group
ITT: intention-to-treat
J2R: jump-to-reference
MAR: missing at random
MICE: multiple imputation by chained equations
MMRM: mixed models for repeated measures
OR: odds ratio
PRAQ: Pregnancy-Related Anxiety Questionnaire
PRAQ-R: Pregnancy-Related Anxiety Questionnaire-Revised
RCT: randomized controlled trial
SCID-5-CV: Structured Clinical Interview for the *Diagnostic and Statistical Manual of Mental Disorders, Fifth Edition*-Clinician Version
SPIRIT: Standard Protocol Items: Recommendations for Intervention Trials
STAI: State-Trait Anxiety Inventory
STAI-S: State-Trait Anxiety Inventory-State
STAI-T: State-Trait Anxiety Inventory-Trait
TAU: treatment as usual
