# Supplementary material for: Digital Mindfulness Intervention for Pregnant Women With Affective Disorders and Acute Stress Reactions: Prespecified Secondary Analysis of a Randomized Controlled Trial
Source: J Med Internet Res. 2026 Sep 2;28:e93811. doi: 10.2196/93811 (PMC13542807; doi:10.2196/93811)
Supplement: Supplementary file 2 [file jmir-v28-e93811-s001.docx]

**Supplemental material**

**eTables**

## eTable 1. Baseline characteristics

eTable 2. Frequency of missing values

eTable 3. MICE-Imputation of the EPDS

eTable 4. Analysis of the STAI-T score (MICE imputation)

eTable 5. Analysis of the STAI-S-Scores (MICE-Imputation)

eTable 6. Pregnancy-related anxiety (PRAQ-R) outcomes

## **eTable 1. Baseline characteristics**

| **Characteristic** | **Control (N = 74)** | **Intervention (N = 73)** |
| --- | --- | --- |
| Age, mean (SD) | 32.5 (4.3) | 31.1 (4.7) |
| Number of children: 0 | 34 (57%) | 31 (53%) |
| 1 | 23 (38%) | 23 (39%) |
| 2 | 2 (3.3%) | 3 (5.1%) |
| 3 | 1 (1.7%) | 1 (1.7%) |
| 4 | 0 (0%) | 1 (1.7%) |
| Missing | 14 | 14 |
| Educational level: No degree | 0 (0%) | 0 (0%) |
| Lower secondary school | 2 (3.3%) | 1 (1.8%) |
| Secondary school | 14 (23%) | 23 (40%) |
| Technical college entrance qualification | 13 (22%) | 10 (18%) |
| High school diploma | 31 (52%) | 23 (40%) |
| Missing | 14 | 16 |
| Household income < €1,500 | 16 (27%) | 16 (28%) |
| €1,500–2,999 | 28 (47%) | 26 (46%) |
| €3,000–4,999 | 14 (23%) | 10 (18%) |
| €5,000–8,000 | 2 (3.3%) | 5 (8.8%) |
| > €8,000 | 0 (0%) | 0 (0%) |
| Missing | 14 | 16 |
| Married, living together | 38 (61%) | 32 (54%) |
| Married, living apart | 1 (1.6%) | 0 (0%) |
| Partnership, living together | 18 (29%) | 20 (34%) |
| Partnership, living apart | 3 (4.8%) | 4 (6.8%) |
| Single | 1 (1.6%) | 3 (5.1%) |
| Divorced | 1 (1.6%) | 0 (0%) |
| Widowed | 0 (0%) | 0 (0%) |
| Missing | 12 | 14 |
| Baseline EPDS score, mean (SD) | 15.5 (3.8) | 15.6 (3.9) |

**eTable 2. Frequency of missing values**

| **Timepoint** | **Control (%)** | **Intervention (%)** |
| --- | --- | --- |
| T0 (Baseline) | 0.0 | 0.0 |
| T1 (28th week of gestation) | 17.1 | 21.0 |
| T2 (30th week of gestation) | 13.1 | 23.3 |
| T3 (32nd week of gestation) | 9.0 | 26.0 |
| T4 (34th week of gestation) | 6.8 | 26.9 |
| T5 (36th week of gestation) | 8.1 | 36.1 |
| T6 (4–6 weeks postpartum) | 40.5 | 48.6 |
| T7 (5 months postpartum) | 33.1 | 43.2 |

**eTable 3. MICE-Imputation of the EPDS**

|  |  |  |  | **95% CI** | |  | **95% CI** | |  |  |  |  |  | **95% CI** | |
| --- | --- | --- | --- | --- | --- | --- | --- | --- | --- | --- | --- | --- | --- | --- | --- |
| **Group** | **Estimate** | **SE** | **df** | **lower** | **upper** | **Difference** | **lower** | **upper** | **SE** | **df** | ***t*** | ***p*** | ***d*** | **lower** | **upper** |
| **T0** | | | | | | | | | | | | | | | |
| control | 15.52 | 0.58 | 142.04 | 14.37 | 16.67 | -0.05 | -1.68 | 1.57 | 0.82 | 142.04 | -0.07 | 0.948 | -0.01 | -0.44 | 0.41 |
| intervention | 15.57 | 0.58 | 142.04 | 14.42 | 16.73 |  |  |  |  |  |  |  |  |  |  |
| **T1** | | | | | | | | | | | | | | | |
| control | 13.54 | 0.66 | 42.90 | 12.20 | 14.88 | 0.08 | -2.11 | 2.27 | 1.05 | 20.52 | 0.07 | 0.942 | 0.02 | -0.59 | 0.63 |
| intervention | 13.46 | 0.89 | 10.29 | 11.49 | 15.44 |  |  |  |  |  |  |  |  |  |  |
| **T2** | | | | | | | | | | | | | | | |
| control | 12.67 | 0.64 | 61.11 | 11.39 | 13.95 | 0.25 | -1.79 | 2.29 | 1.00 | 28.24 | 0.25 | 0.801 | 0.07 | -0.46 | 0.59 |
| intervention | 12.41 | 0.69 | 34.47 | 11.02 | 13.81 |  |  |  |  |  |  |  |  |  |  |
| **T3** | | | | | | | | | | | | | | | |
| control | 12.80 | 0.58 | 138.89 | 11.65 | 13.96 | 1.72 | -0.19 | 3.62 | 0.94 | 42.63 | 1.82 | 0.076 | 0.45 | -0.06 | 0.96 |
| intervention | 11.09 | 0.73 | 23.21 | 9.58 | 12.60 |  |  |  |  |  |  |  |  |  |  |
| **T4** | | | | | | | | | | | | | | | |
| control | 12.38 | 0.62 | 83.12 | 11.15 | 13.62 | 2.21 | 0.49 | 3.93 | 0.87 | 97.51 | 2.54 | 0.013 | 0.57 | 0.12 | 1.03 |
| intervention | 10.17 | 0.64 | 67.53 | 8.90 | 11.45 |  |  |  |  |  |  |  |  |  |  |
| **T5** | | | | | | | | | | | | | | | |
| control | 12.67 | 0.59 | 133.97 | 11.51 | 13.84 | 3.25 | 0.79 | 5.70 | 1.14 | 14.04 | 2.84 | 0.013 | 0.84 | 0.13 | 1.55 |
| intervention | 9.43 | 1.06 | 6.97 | 6.93 | 11.93 |  |  |  |  |  |  |  |  |  |  |
| **T6** | | | | | | | | | | | | | | | |
| control | 12.70 | 0.79 | 15.01 | 11.01 | 14.38 | 4.81 | 1.68 | 7.95 | 1.37 | 8.28 | 3.52 | 0.007 | 1.25 | 0.47 | 2.03 |
| intervention | 7.88 | 0.83 | 13.06 | 6.10 | 9.67 |  |  |  |  |  |  |  |  |  |  |
| **T7** | | | | | | | | | | | | | | | |
| control | 12.00 | 0.94 | 8.82 | 9.87 | 14.12 | 2.38 | -1.61 | 6.36 | 1.63 | 6.02 | 1.46 | 0.195 | 0.62 | -0.35 | 1.58 |
| intervention | 9.62 | 0.92 | 9.42 | 7.55 | 11.69 |  |  |  |  |  |  |  |  |  |  |

**eTable 4. Analysis of the STAI-T score (MICE imputation)**

|  |  |  |  | **95% CI** | |  | **95% CI** | |  |  | |  | |  | |  | | **95% CI** | |
| --- | --- | --- | --- | --- | --- | --- | --- | --- | --- | --- | --- | --- | --- | --- | --- | --- | --- | --- | --- |
| **Group** | **Estimate** | **SE** | **df** | **lower** | **upper** | **Difference** | **lower** | **upper** | **SE** | | **df** | | ***t*** | | ***p*** | | ***d*** | **lower** | **upper** |
| **T1** | | | | | | | | | | | | | | | | | | | |
| control | 53.27 | 1.14 | 140.59 | 51.02 | 55.52 | 0.70 | -2.51 | 3.91 | 1.62 | | 138.63 | | 0.43 | | 0.668 | | 0.08 | -0.27 | 0.42 |
| intervention | 52.58 | 1.15 | 138.80 | 50.30 | 54.85 |  |  |  |  | |  | |  | |  | |  |  |  |
| **T2** | | | | | | | | | | | | | | | | | | | |
| control | 52.43 | 1.33 | 34.98 | 49.73 | 55.13 | -0.31 | -4.19 | 3.57 | 1.91 | | 32.92 | | -0.16 | | 0.872 | | -0.03 | -0.45 | 0.39 |
| intervention | 52.74 | 1.34 | 35.50 | 50.03 | 55.45 |  |  |  |  | |  | |  | |  | |  |  |  |
| **T3** | | | | | | | | | | | | | | | | | | | |
| control | 50.76 | 1.18 | 112.86 | 48.43 | 53.09 | 1.09 | -2.52 | 4.71 | 1.80 | | 52.85 | | 0.61 | | 0.546 | | 0.12 | -0.28 | 0.52 |
| intervention | 49.66 | 1.39 | 26.61 | 46.80 | 52.52 |  |  |  |  | |  | |  | |  | |  |  |  |
| **T4** | | | | | | | | | | | | | | | | | | | |
| control | 51.39 | 1.22 | 81.23 | 48.98 | 53.81 | 5.25 | 1.70 | 8.79 | 1.77 | | 61.41 | | 2.96 | | 0.004 | | 0.57 | 0.17 | 0.97 |
| intervention | 46.15 | 1.35 | 33.60 | 43.41 | 48.88 |  |  |  |  | |  | |  | |  | |  |  |  |
| **T5** | | | | | | | | | | | | | | | | | | | |
| control | 50.31 | 1.18 | 105.74 | 47.96 | 52.66 | 3.63 | -0.34 | 7.59 | 1.94 | | 29.17 | | 1.87 | | 0.071 | | 0.39 | -0.06 | 0.84 |
| intervention | 46.68 | 1.55 | 15.40 | 43.39 | 49.97 |  |  |  |  | |  | |  | |  | |  |  |  |
| **T6** | | | | | | | | | | | | | | | | | | | |
| control | 49.82 | 1.63 | 12.21 | 46.27 | 53.37 | 6.07 | 0.78 | 11.36 | 2.40 | | 10.94 | | 2.53 | | 0.028 | | 0.66 | 0.09 | 1.23 |
| intervention | 43.75 | 1.61 | 13.20 | 40.27 | 47.22 |  |  |  |  | |  | |  | |  | |  |  |  |
| **T7** | | | | | | | | | | | | | | | | | | | |
| Kontrolle | 49.75 | 1.73 | 10.16 | 45.90 | 53.61 | 4.39 | -0.47 | 9.25 | 2.26 | | 13.48 | | 1.94 | | 0.073 | | 0.48 | -0.07 | 1.03 |
| Intervention | 45.36 | 1.31 | 43.23 | 42.73 | 48.00 |  |  |  |  | |  | |  | |  | |  |  |  |

**eTable 5. Analysis of the STAI-S-Scores (MICE-Imputation)**

|  |  |  |  | 95% CI | |  | 95% CI | |  |  |  |  |  | 95% CI | |
| --- | --- | --- | --- | --- | --- | --- | --- | --- | --- | --- | --- | --- | --- | --- | --- |
| Group | Estimate | SE | df | lower | upper | Difference | lower | upper | SE | df | *t* | *p* | *d* | lower | upper |
| T1 | | | | | | | | | | | | | | | |
| control | 52.52 | 1.24 | 131.81 | 50.07 | 54.98 | 0.74 | -2.79 | 4.27 | 1.78 | 121.73 | 0.42 | 0.679 | 0.07 | -0.27 | 0.41 |
| intervention | 51.78 | 1.28 | 107.28 | 49.24 | 54.33 |  |  |  |  |  |  |  |  |  |  |
| T2 | | | | | | | | | | | | | | | |
| control | 51.93 | 1.27 | 112.46 | 49.42 | 54.45 | 0.36 | -3.40 | 4.12 | 1.89 | 70.44 | 0.19 | 0.848 | 0.03 | -0.32 | 0.39 |
| intervention | 51.57 | 1.35 | 64.21 | 48.88 | 54.27 |  |  |  |  |  |  |  |  |  |  |
| T3 | | | | | | | | | | | | | | | |
| control | 50.47 | 1.31 | 81.25 | 47.87 | 53.08 | 1.48 | -2.30 | 5.26 | 1.89 | 67.12 | 0.78 | 0.438 | 0.14 | -0.23 | 0.51 |
| intervention | 49.00 | 1.43 | 37.24 | 46.09 | 51.90 |  |  |  |  |  |  |  |  |  |  |
| T4 | | | | | | | | | | | | | | | |
| control | 52.00 | 1.34 | 63.79 | 49.32 | 54.67 | 4.39 | 0.26 | 8.52 | 2.04 | 35.18 | 2.16 | 0.038 | 0.42 | 0.02 | 0.82 |
| intervention | 47.60 | 1.51 | 25.97 | 44.51 | 50.70 |  |  |  |  |  |  |  |  |  |  |
| T5 | | | | | | | | | | | | | | | |
| control | 51.73 | 1.25 | 124.45 | 49.25 | 54.21 | 4.71 | 1.00 | 8.42 | 1.86 | 78.80 | 2.53 | 0.014 | 0.45 | 0.09 | 0.81 |
| intervention | 47.02 | 1.39 | 49.73 | 44.23 | 49.80 |  |  |  |  |  |  |  |  |  |  |
| T6 | | | | | | | | | | | | | | | |
| control | 47.72 | 1.41 | 39.37 | 44.87 | 50.58 | 5.63 | 1.94 | 9.32 | 1.86 | 82.59 | 3.03 | 0.003 | 0.54 | 0.15 | 0.92 |
| intervention | 42.09 | 1.71 | 13.96 | 38.43 | 45.76 |  |  |  |  |  |  |  |  |  |  |
| T7 | | | | | | | | | | | | | | | |
| control | 46.68 | 1.30 | 84.49 | 44.08 | 49.27 | 1.65 | -2.29 | 5.60 | 1.96 | 48.19 | 0.84 | 0.403 | 0.16 | -0.23 | 0.55 |
| intervention | 45.02 | 1.60 | 18.73 | 41.67 | 48.37 |  |  |  |  |  |  |  |  |  |  |

### **eTable 6.** **Pregnancy-related anxiety (PRAQ-R) outcomes**

|  |  |  |  | **95% CI** | |  | **95% CI** | |  |  |  |  |  | **95% CI** | |
| --- | --- | --- | --- | --- | --- | --- | --- | --- | --- | --- | --- | --- | --- | --- | --- |
| **Group** | **Estimate** | **SE** | **df** | **lower** | **upper** | **Difference** | **lower** | **upper** | **SE** | **df** | ***t*** | ***p*** | ***d*** | **lower** | **upper** |
| T1 | | | | | | | | | | | | | | | |
| control | 27.39 | 0.61 | 141.50 | 26.19 | 28.60 | 0.20 | -1.51 | 1.92 | 0.87 | 140.17 | 0.23 | 0.816 | 0.03 | -0.19 | 0.25 |
| intervention | 27.19 | 0.62 | 138.54 | 25.97 | 28.41 |  |  |  |  |  |  |  |  |  |  |
| T2 | | | | | | | | | | | | | | | |
| control | 27.53 | 0.67 | 58.69 | 26.18 | 28.87 | -0.43 | -2.41 | 1.56 | 0.99 | 44.33 | -0.43 | 0.667 | -0.05 | -0.32 | 0.21 |
| intervention | 27.95 | 0.75 | 26.08 | 26.41 | 29.50 |  |  |  |  |  |  |  |  |  |  |
| T3 | | | | | | | | | | | | | | | |
| control | 26.52 | 0.77 | 21.70 | 24.92 | 28.11 | -0.76 | -3.08 | 1.56 | 1.11 | 19.72 | -0.68 | 0.504 | -0.10 | -0.40 | 0.20 |
| intervention | 27.27 | 0.79 | 18.95 | 25.61 | 28.94 |  |  |  |  |  |  |  |  |  |  |
| T4 | | | | | | | | | | | | | | | |
| control | 27.56 | 0.70 | 42.67 | 26.15 | 28.97 | 1.37 | -0.73 | 3.47 | 1.03 | 31.22 | 1.33 | 0.193 | 0.18 | -0.13 | 0.49 |
| intervention | 26.19 | 1.06 | 7.55 | 23.71 | 28.66 |  |  |  |  |  |  |  |  |  |  |
| T5 | | | | | | | | | | | | | | | |
| control | 26.96 | 0.78 | 20.47 | 25.34 | 28.58 | 0.43 | -3.35 | 4.21 | 1.59 | 6.78 | 0.27 | 0.793 | 0.06 | -0.44 | 0.55 |
| intervention | 26.53 | 1.17 | 6.34 | 23.70 | 29.36 |  |  |  |  |  |  |  |  |  |  |
